# Synthesis of Quinolones and Zwitterionic Quinolonate Derivatives with Broad-Spectrum Antibiotic Activity

**DOI:** 10.3390/ph15070818

**Published:** 2022-07-01

**Authors:** Beatriz Suay-García, Jose-Ignacio Bueso-Bordils, Gerardo Antón-Fos, María-Teresa Pérez-Gracia, Antonio Falcó, Pedro Alemán-López

**Affiliations:** 1 ESI International Chair@CEU-UCH, Departamento de Matemáticas, Física y Ciencias Tecnológicas, Universidad Cardenal Herrera-CEU, CEU Universities San Bartolomé 55, 46115 Alfara del Patriarca, Valencia, Spain; afalco@uchceu.es; 2 Departamento de Farmacia, Universidad Cardenal Herrera-CEU, CEU Universities C/Ramón y Cajal s/n, 46115 Alfara del Patriarca, Valencia, Spain; jose.bueso@uchceu.es (J.-I.B.-B.); ganton@uchceu.es (G.A.-F.); teresa@uchceu.es (M.-T.P.-G.)

**Keywords:** quinolone, antibiotic, antibiotic-resistant bacteria, zwitterionic compounds

## Abstract

Quinolones are one of the most extensively used therapeutic families of antibiotics. However, the increase in antibiotic-resistant bacteria has rendered many of the available compounds useless. After applying our prediction model of activity against *E. coli* to a library of 1000 quinolones, two quinolones were selected to be synthesized. Additionally, a series of zwitterionic quinolonates were also synthesized. Quinolones and zwitterionic quinolonates were obtained by coupling the corresponding amine with reagent **1** in acetonitrile. Antibacterial activity was assessed using a microdilution method. All the compounds presented antibacterial activity, especially quinolones **2** and **3**, selected by the prediction model, which had broad-spectrum activity. Furthermore, a new type of zwitterionic quinolonate with antibacterial activity was found. These compounds can lead to a new line of antimicrobials, as the structures, and, therefore, their properties, are easily adjustable in the amine in position 4 of the pyridine ring.

## 1. Introduction

The discovery and synthesis of antibiotic compounds was a turning point in the treatment of infectious diseases. However, the extreme versatility and adaptability of microorganisms has prevented a decrease in the prevalence of these diseases, seeing as bacteria have developed mechanisms that make them resistant to these drugs [1]. The threat of antibiotic-resistant bacteria is such that the World Health Organization (WHO) has announced that, if resistance continues to increase at the current rate, infections caused by antibiotic-resistant bacteria will become the leading cause of death worldwide, exceeding deaths caused by cancer, diabetes and cardiovascular diseases [2].

Within the different antibiotic families being currently used in clinical practice, quinolones are one of the most extensively used therapeutic families in the treatment of infectious diseases. In fact, this group gained clinical relevance due to antibiotic-resistant bacteria, since, during the 1980s, it was used to treat infections caused by *Shigella* spp. and *Escherichia coli* strains resistant to other available antibacterial drugs [3]. The pharmacokinetic profile of quinolones, along with their high antibacterial activity and their broad spectrum, result in quinolones having many therapeutic indications, both in hospitals and community settings [4]. However, to avoid the appearance of resistance, these drugs should have been limited to situations where the microorganism presents multi-resistance or there are contraindications for the use of other antibacterial agents.

The development of this antibacterial family is based on the possibility of performing substitutions in all the positions, except for 3 and 4, which must remain constant. These variations affect the selectivity and efficacy of quinolones against different microorganisms and modify pharmacokinetic properties, toxicity and interaction with other drugs [5]. Along these lines, QSAR methods are an efficient tool to carry out virtual screenings to identify antibacterial drug candidates [6]. Furthermore, this chemoinformatic approach allows the prediction of pharmacokinetic and toxicological properties directly related to activity to identify safer and more effective drugs [6].

**Figure 1:**
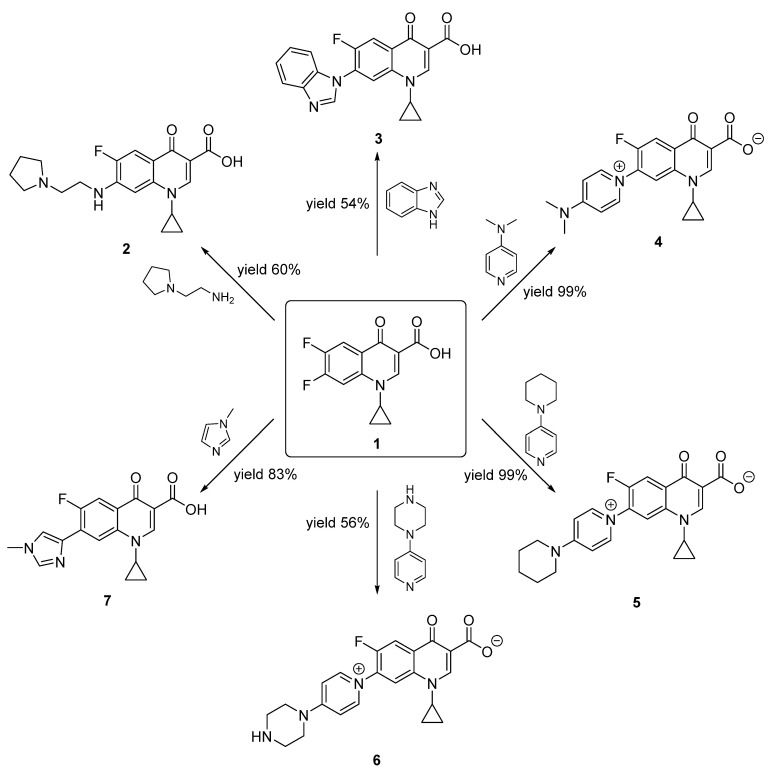
Quinolones and zwitterionic quinolonates synthesized from the commercial difluorinated quinolone **1**.

The aim of this paper is to synthesize new quinolones selected by a prediction model and evaluate their antibiotic activity. Furthermore, three new zwitterionic quinolonate compounds and quinolone **7** were also synthesized and their antibacterial activity was assessed. All these compounds were synthesized using a one-step reaction between the commercial difluorinated quinolone **1** and the corresponding amine (Figure 1).

## 2. Results

### 2.1. Compound Selection

**Figure 2:**
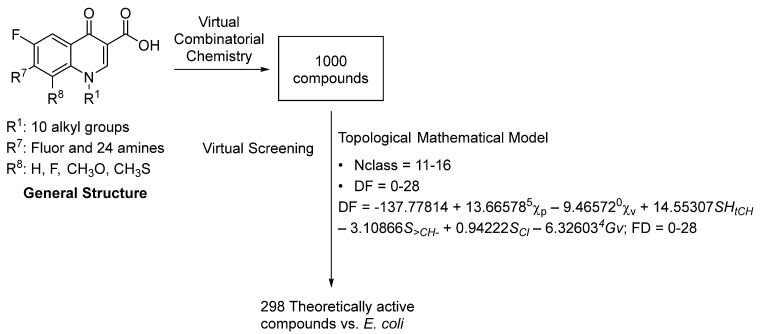
Virtual combinatorial chemistry and pharmacological screening to find new quinolones with theoretical antibacterial activity.

A mathematical–topological prediction model previously described by our research group was used to select theoretically active compounds against E. coli from a virtual combinatorial library of 1000 quinolones [7,8]. In total, 298 quinolones from the virtual combinatorial library were selected by the model as theoretically active against *E. coli* (Figure 2) (see Supplementary Materials).

As the model established, the selected molecules had a value for index Nclass between 11 and 16 and a value for the DF within the 0–28 range [7]. Nclass is a topological index that could be defined as the topological distance (number of axes) between the two furthest vertices of a graph through the shortest path [7]. Molecules containing fluorine in position 7 were not selected as their value for Nclass is < 11 (except for two, all have values for the DF between 0 and 28), which implies that the theoretically active compounds must have a substituent in position 7 with a certain degree of structural complexity. Practically all of the used amines appear within the selected molecules. Regarding the simplest amine, cyclopropylamine, the only compounds selected were those with a benzyl substituent in position 1, seeing as this substituent provides size and complexity, which increases its Nclass value. As for the substituents in positions 1 and 8, no priority was observed among them as all appear in similar proportions in the selected molecules.

**Figure 3:**
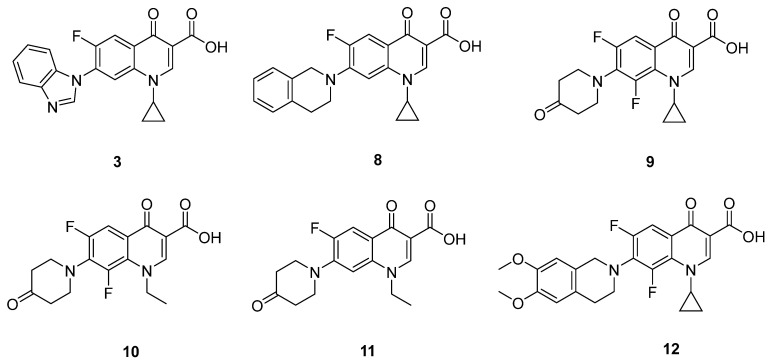
Quinolones selected by the model that had been described in the literature as active antimicrobials.

In terms of pharmacological activity, six of the quinolones selected by the prediction model have been described in the literature (Figure 3). Those six quinolones correspond to: compound **3**, which was synthesized and assayed against methicillin-resistant *Staphylococcus aureus* (MRSA) by our research group [8], and compounds **8**, **9**, **10**, **11** and **12** [9,10,11,12], which had already been assayed against *E. coli* with minimum inhibitory concentrations (MICs) between 0.5 and 2 µg/mL. These data validate the ability of our model to find new molecules with antibiotic activity.

**Figure 4:**
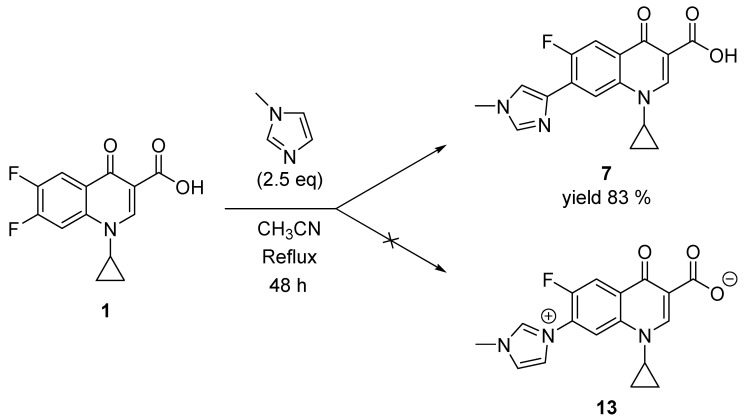
Obtention of **7** as the only product of the reaction between **1** and 1-methylimidazole.

Quinolones **2** and **3** were selected to be synthesized by reacting the difluorinated quinolone **1** with the corresponding amine (Table 1) [8]. Furthermore, their antibacterial activity was assessed against different microorganisms. Quinolone **7**, which was obtained unexpectedly in the reaction between 1-methylimidazole and compound **1** (Figure 4), was also assayed against a battery of microorganisms.

Additionally, three new zwitterionic quinolonate compounds were also selected to be synthesized and have their antibacterial activity assayed. As will be detailed later on, these compounds appeared during the study of the possibility of accelerating the reaction between **1** and benzymidazole with 4-dimethylaminopyridine (DMAP).

Zwitterionic compounds containing quaternary amines are becoming increasingly popular as new antibiotics due to the ease with which products can be prepared to have a cationic character in the infection site with acidic pH, imitating natural antibacterial peptides [13,14].

Moreover, pyridinium and imidazolium salts have also become popular for being ionic liquids [15]. Different studies show the negative effect these compounds can have on the environment, as well as their toxicological effects in different microorganisms [16,17]. According to Riduan et al. [18], the toxicity of these salts could be due to their amphipathic structure, in which the hydrophilic cation interacts with biological components in the cell membrane or DNA, while the hydrophobic segment causes structural and functional disruptions. Modelling of the toxicity of these pyridinium and imidazolium salts could result in products with interesting applications for humans, such as the design of new antimicrobial agents.

Several studies prove the antibacterial activity of pyridinium salts. One example is that of Li et al. [19], in which they synthesized pyridinium *N*-chloramines with activity against *E. coli* and *S. aureus*. Similarly, Alptunzu et al. [20] synthesized several benzylidenhydrazineilpiridinium salts. Some of them showed activity against *S. aureus* with an MIC in the 4–8 μg/mL range. A group of chalcone–pyridinium hybrids also presented antibacterial and antitumoral activity similar to that of streptomycin [21].

For these reasons and due to the ease with which compound **4** was obtained, we decided to synthesize structural analogues **5** and **6.**

Similar to pyridinium salts, there are numerous studies that point out the potential of imidazolium salts as antimicrobials [22,23,24]. For this reason, the synthesis of quinolonate **13** was planned replacing DMAP with 1-methylimidazole but quinolone **7** was unexpectedly obtained as the only reaction product (Figure 4). Therefore, its activity was also assayed. Testing the activity of secondary products during the synthesis of a compound is a common practice. In fact, the first antimicrobial quinolone was discovered as an impurity in the chemical manufacture of chloroquine [10].

### 2.2. Quinolone Synthesis

Quinolone **2** was obtained by coupling the corresponding amine with reagent **1** in acetonitrile, following well-established research [25]. The reaction was followed using thin-layer chromatography and, after 3 h, reagent **1** had been completely used up. The mixture was concentrated and, after adding water, the formed precipitate was filtered under high vacuum. Furthermore, the filtration liquid was treated with HCl to adjust its pH to 7, which resulted in the formation of a new solid that was also filtered under high vacuum. Quinolone **2** was obtained with a 60% yield (Table 1, entry 1).

On the other hand, the obtention of compound **3** by reacting **1** with benzimidazole required long reaction times [8]. After 10 days of reaction, the yield was only 18% (Table 1, entry 2) and, due to the great amount of starting reagent remaining, the purification had to be carried out using preparative thin-layer chromatography with CH_2_Cl_2_:CH_3_OH 20:1 as eluent. Along these lines, Gellis et al. [26] described that the substitution reaction of chlorine for amine in position 4 of the quinazoline ring is catalyzed by DMAP. Consequently, we decided to study the possibility of DMAP catalyzing the reaction. When the reaction was carried out adding a catalytic amount of DMAP, the yield increased to 54% in the same reaction time (Table 1, entry 3). Moreover, by increasing the proportion of product in the reaction mixture, the purification could be carried out through simple filtration and washing. Assuming that the catalytic capacity of DMAP is due to its nucleophilic properties, we decided to also try triphenylphosphine as a catalyst. In this case, we found no improvement in the yield and, after 10 days of reaction, product **3** was obtained with a 17% yield (Table 1, entry 4).

### 2.3. Zwitterionic Quinolonate Synthesis

Compound **4** was obtained quantitatively by reacting compound **1** with an excess of DMAP in reflux with acetonitrile (Table 2, entry 1). The same synthetic methodology was used to obtain compounds **5** and **6**, by reacting **1** with 4-(piperidin-1-yl)pyridine and 1-(pyridin-4-yl)piperazine, respectively (Table 2, entries 2 and 3). These reagents were selected because they include piperidine and piperazine rings while barely altering the reactivity of the pyridine’s nitrogen. Piperidine and piperazine scaffolds are included in many drug structures [27,28].

To know if this reaction was extensible to imidazolic derivatives, DMAP was substituted with 1-methylimidazole to obtain compound **13** following the same procedure. However, the reaction did not occur through the imidazole nitrogen; instead, it occurred through the carbon 4 of the imidazole ring. Compound **7** was the only product observed in the reaction and was obtained with an 83% yield (Figure 4).

### 2.4. Antibiotic Activity (MIC and MBC)

The antibiotic activity assays of compounds **2** and **3** are shown in Table 3, including MIC and MBC after 18–24 h of incubation at 37 °C. Ciprofloxacin was used as the reference antibacterial drug in order to compare the activity of the synthesized quinolones.

Seeing the activity values of both quinolones against *E. coli*, we decided to study the antimicrobial activity spectrum of compounds **2** and **3** against Gram-positive and Gram-negative bacteria, as well as against *Candida albicans* (Table 4).

Additionally, the antibacterial activity of the synthesized zwitterionic quinolonates and quinolone **7** was also assessed (Table 5).

## 3. Discussion

DMAP is a reagent that can act as a nucleophilic catalyst in many transformations [29]. We observed that the reaction of reagent 1 with benzimidazole to obtain quinolone **3** could be catalyzed by DMAP (Table 1, entries 1 and 2), as the addition of this catalyst leads to a yield increase in the same reaction time.

**Figure 5:**
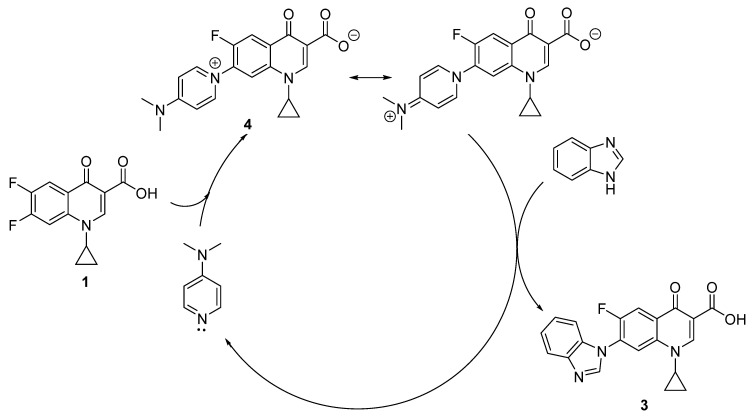
Probable mechanism for the obtention of **3** catalyzed by DMAP.

Analogously to the mechanism postulated by Gellis et al., DMAP may react with the difluorinated quinolone **1** to produce the intermediate **4** that would then react with benzimidazole to result in compound **3** and DMAP would be regenerated to participate in the next catalytic cycle (Figure 5). To support this hypothesis, in the same way Gellis et al. did, we decided to synthesize our zwitterionic intermediate **4**, which was obtained quantitatively by reacting **1** with an excess DMAP (Table 2, entry 1).

Using the same methodology, treating **1** with an excess of 4-(piperidin-1-yl)pyridine quantitatively precipitated **5** (Table 2, entry 2). However, compound **6** was obtained with a 56% yield. In this case, reagent **1** was also completely used up but the chromatography of the precipitated crude reaction showed that compound **6** had an impurity with lower polarity. Said impurity was eliminated through successive extractions with warm acetonitrile. Mass spectrometry analysis of the impurity showed that it was a compound with molecular formula C_27_H_22_O_4_, eliminating any possibility of it being the quinolone that would result from an attack of the piperazinic nitrogen.

Lastly, the reaction with 1-methylimidazole did not evolve as expected to obtain the corresponding zwitterionic compound. The reaction was slower than with the rest of the amines, seeing as, after 48 h, there was one single product and some reagent **1** left. We decided to stop the reaction and compound **7** was obtained with an 83% yield. When trying to carry out the spectroscopic assignment of the obtained compound, we found that the resulting product was not the expected imidazolium quinolonate **13**.

Instead, compound **7** was obtained unexpectedly (Figure 5). The mass spectrometry analysis came out as expected, revealing a compound with molecular formula C_17_H_14_FN_3_O_3_. However, only five aromatic protons were observed in the ^1^H NMR, which indicates that the reaction occurred through one of the imidazolic carbons. The reactions of electrophilic C-substitution in the imidazole are difficult to predict. Nitrations and halogenations preferably occur in position C-4 or positions C-4 and C-5 [30]. Reactions of aldehydes and acid chlorides have also been described in position C-2 [31]. The addition in C-2 was rejected because hydrogens in positions C-4 and C-5 of the *N*-alkylimidazolic derivatives substituted in C-2 usually appear at 7 and 6.9 ppm, respectively [32] and our compound shows two imidazolic hydrogens at 7.9 and 7.7 ppm, which correspond to the hydrogens in positions 2 and 5, respectively.

Seeing as **7** had a quinolone structure, its antibacterial activity was also assayed.

In terms of the antibacterial activity of the synthesized quinolones, compounds **2** and **3** displayed antibacterial activity against *E. coli*, seeing as both had an MIC value within the sensibility range, established by CLSI guidelines [33]. It must be noted that compound **3** showed a lower MBC (32 mg/L) than ciprofloxacin (64 mg/L) (Table 3).

Furthermore, both quinolones displayed broad-spectrum antimicrobial activity (Table 4) as they presented low-MIC values against Gram-positive and Gram-negative bacteria. More specifically, compound **3** stands out for its high activity against Gram-positive bacteria, reaching an MIC under 0.03 mg/L against *B. subtilis* (Table 4), which proves it has the same activity as ciprofloxacin (MIC 0.05 mg/L) [34]. It should also be noted that compound **3** is more active than ciprofloxacin against MRSA, as described by Bueso-Bordils et al. [8]. Moreover, this quinolone presents high activity against Gram-negative bacteria, with MICs within the range established by CLSI for quinolones against *P. aeruginosa* and *E. coli*, 2 mg/L.

Quinolone **2**, while active against Gram-positive and Gram-negative bacteria, must be highlighted for its moderate activity against *C. albicans*. Other studies reported activity of other commercial quinolones, such as moxifloxacin, gatifloxacin and sparfloxacin, against *C. albicans* [35,36]. Topoisomerase II has been identified as the main target for quinolones against yeasts [37], which is more susceptible to quinolones in yeasts than in mammals [38]. Therefore, it can be considered that the two synthesized quinolones (compounds **2** and **3**) have a broad antimicrobial activity spectrum.

Regarding the antibacterial activity of the zwitterionic quinolonates, Table 5 shows that compounds **4**, **5** and **6** have moderate activity against Gram-positive bacteria. Furthermore, the activity of compound **4** against *E. coli* must also be noted. Thus, these zwitterionic quinolonates could be interesting lead compounds for the development of new antibacterial families. On another note, compound **7** showed activity against *E. coli*.

## 4. Materials and Methods

### 4.1. Synthesis

The solvents used were dried following described procedures [39]. The reagents were obtained from commercial suppliers (Aldrich, Fluka and Scharlaud) and did not undergo any prior purification. The solid reagents were dried before using them by keeping them in a vacuum in the presence of P_2_O_5_ for, at least, 12 h. The triethylamine used was distilled at the time it was going to be used, in an argon atmosphere with sodium hydroxide pellets [39].

The thin-layer chromatography (TLC) analyses were carried out in silica gel (Kieselgel 60 F254 on plastic) active under ultraviolet (UV) light and were visualized using 254 nm and/or 365 nm UV light. Iodine was also used as a developer.

The fusion point was determined using a “Cambridge Instruments” machine; it has not been corrected and is only indicative.

The infrared (IR) spectrum was obtained using the Spectrum Two FT-IR spectrometer and the Universal ATR diamond polarization accessory.

The ^1^H NMR spectra were obtained with a Brucker 300 spectrophotometer, using deuterated chloroform (CDCl_3_), deuterated dimethylsulfoxide (DMSO-d6), deuterated methanol (CD_3_OD) or deuterium oxide (D_2_O) as a solvent. The nuclei chemical shift values are expressed in ∂ values (ppm).

A hybrid mass spectrometer with quadruple time-of-fly analyzer was used to determine mass spectra (TRIPLETOFT5600, ABSciex). The conditions were: direct infusion, electrospray technique (ESI), ion source gas 1 (GC1): 30 psi, ion source gas 2 (GC2): 30 psi, curtain gas 1: 25 psi, 450 °C, ion spray voltage (ISVF): 5500, mass range: 80–950 *m*/*z*.

Compound purity was confirmed by a combination of LC-MS (HPLC) and high-resolution mass spectrometry and NMR analysis. All compounds had >95% purity.

#### 4.1.1. Quinolone Synthesis

1-cyclopropyl-6-fluoro-4-oxo-7-((2-(pyrrolidine-1-il) ethyl)amino)-1,4-dihydroquinoline-3-carboxylic acid (**2**)

A mixture of 1 (209 mg, 0.79 mmol), (2-aminoethyl)pyrrolidine (361 mg, 3.16 mmol) and 8.5 mL of anhydrous CH_3_CN was refluxed with drying tube. After 3 h, the mixture was concentrated; then, 2 mL of water was added, resulting in the formation of a precipitate which was filtered and washed with cold water and dried under high vacuum. This solid corresponded to 73 mg of **2**. Furthermore, the filtration liquid was treated with HCl to adjust its pH to 7, which resulted in the formation of a new solid that was filtered and washed with cold water and dried under high vacuum obtaining 97 mg of **2**. Pure fluoroquinolone **2** was obtained with an overall yield of 60% (170 mg) as an off-white solid, mp 179.8 °C. IR (cm^−1^): 3344.4; 3082.7; 3026.7; 2964.2; 2939.5; 2877.0; 1724.0; 1633.6; 1526.1; 1455.3; 1396.8. NMR 1H (300 MHz, CDCl3) δ: 15.88–14.83 (br, 1H), 8.61(s, 1H), 7.84 (d, J = 11.6 Hz, 1H), 6.90 (d, J = 7.1 Hz, 1H), 5.56–5.45 (m, 1H), 3.45 (tt, J = 7.2, 4.0 Hz, 1H), 3.36–3.23 (m, 2H), 2.81 (t, J = 6.1 Hz, 2H), 2.60–2.49 (m, 4H), 1.79–1.75 (m, 4H) and 1.35–1.07 (m, 4H). HRMS (ESI-TOF) *m*/*z*: (M + H)^+^ calculated for C_19_H_22_FN_3_O_3_ 360.1718, found: 360.1725.

7-(1H-benzo[d]imidazol-1-yl)-1-cyclopropyl-6-fluoro-4-oxo-1,4-dihydroquinoline-3-carboxylic acid (**3**)

**Without a catalyst:**

A mixture of 1 (200 mg, 0.754 mmol), benzimidazole (177 mg, 1.5 mmol), anhydrous CH_3_CN (11 mL) and Et_3_N (0.41 mL, 3 mmol) was refluxed with drying tube. After 240 h, the resulting suspension was filtered and washed first with CH_3_CN (3 **×** 3 mL) and later with water (3 × 3 mL). The solid was purified by PTLC with a mixture CH_2_Cl_2_:CH_3_OH 20:1 as eluent. Compound **3** (49 mg, 18%) was obtained as an off-white solid.

**With a catalyst:**

A mixture of **1** (200 mg, 1.5 mmol), benzimidazole (295 mg, 2.5 mmol), 8.5 mL of anhydrous CH_3_CN and Et_3_N (0.94 µL, 6.7 mmol) and the corresponding catalyst, 0.3 mmol DMAP, was refluxed with drying tube. The white suspension was filtered and the solid was washed first with warm CH_3_CN and then with cold water. Compound **3** (148 mg, 54%) was obtained as an off-white solid, mp: 292–293 °C. IR (cm^−1^): 3066.64; 1712.91; 1616.99; 1504.51; 1479.92; 1447.08. NMR ^1^H (300 MHz, DMSO-d6) δ: 14.70 (s, 1H); 8.85 (s, 1H); 8.75–8.60 (m, 2H); 8.39 (d; J = 10.4 Hz; 1H); 7.92–7.79 (m, 1H); 7.67–7.57 (m, 1H); 7.49–7.30 (m, 2H); 3.99–3.84 (br s, 1H); and 1.35–1.20 (m, 4H). HRMS (ESI-TOF) *m*/*z*: (M + H)^+^ calculated for C_20_H_14_FN_3_O_3_ 364.1092. Found 364.1108.

#### 4.1.2. Zwitterionic Quinolonate Synthesis

A mixture of **1** (150 mg, 0.567 mmol), 2.5 or 3.5 equivalents of the corresponding amine (Table 2) and 8.5 mL of anhydrous CH_3_CN was refluxed with drying tube. After the time indicated in Table 2 had passed, the resulting mixtures were filtered and the solid was washed with warm acetonitrile (3 × 3 mL). After drying the product under vacuum:

1-cyclopropyl-7-[4-(dimethylamino)pyridinium-1-yl]-6-fluoro-4-oxo-1,4-dihydroquinoline-3-carboxilate (**4**)

Here, 206 mg of white solid was obtained (yield: 99%; m.p.: >350 °C). IR (cm^−1^): 3096.64; 3068.54; 3047.37; 2882.85; 1703.56; 1650.09; 1587.35; 1474.30; 1167.87. NMR ^1^H (300 MHz, CD_3_OD) δ: 8.70 (s, 1H); 8,49 (d; *J* = 5.6 Hz; 1H); 8.38 (d; *J* = 5.6 Hz; 2H); 8.16 (d; *J* = 10.3 Hz; 1H); 7.16 (d; *J* = 6.0 Hz; 2H); 3.86–3.56 (br, 1H); 3.30 (s, 6H); and 1.40–1.06 (m, 4H). HRMS (ESI-TOF) *m*/*z*: (M + H)^+^ calculated for C_20_H_18_FN_3_O_3_ 368.1405, found 368.1404.

1-cyclopropyl-6-fluoro-4-oxo-7-(4-(piperidin-1-yl)pyridin-1-ium-1-yl)-1,4-dihydroquinoline-3-carboxylate (**5**)

Here, 229 mg of white solid was obtained (yield: 99%; m.p.: 229–233 °C). IR (cm^−1^): 3375.12, 3084.20, 3069.40, 2952.30, 1643.23, 1617.69, 1561.62, 1483.47. NMR ^1^H (300 Hz, CD_3_OD) δ: 8.65–8.45 (br, 2H), 8.47 (s, 1H), 8.31 (s, 1H), 7.94 (d, *J* = 10.5 Hz, 1H), 7.35 (d, *J* = 5.2 Hz, 2H), 3.87 (t, *J* = 4.9 Hz, 4H), 3.80–3.50 (m, 1H), 2.02–1.62 (m, 6H), and 1.45–1.02 (m, 4H). HRMS (ESI-TOF) *m*/*z*: (M + H)^+^ calculated for C_23_H_22_FN_3_O_3_ 408.1718, found 408.1713.

1-cyclopropyl-6-fluoro-4-oxo-7-(4-(piperazin-1-yl)pyridin-1-ium-1-yl)-1,4-dihydroquinoline-3-carboxilate (**6**)

Here, 130 mg of off-white solid was obtained (yield: 56%; m.p.: >350 °C). IR (cm^−1^): 3377.06, 3205.10, 3092.6, 1615.01, 1556.48, 1481.59. NMR ^1^H (300 Hz, D_2_O) δ: 8.49 (s, 1H), 8.43–8.26 (m, 3H), 8.04 (d, *J* = 10.7 Hz, 1H), 7.25 (d, *J* = 7.4 Hz, 2H), 3.93 (t, *J* = 5.3 Hz, 4H), 3.70–3.50 (m, 1H), 3.26 (t, *J* = 5.3 Hz, 4H), and 1.34–1.03 (m, 4H). HRMS (ESI-TOF) *m*/*z*: (M + H)^+^ calculated for C_22_H_21_FN_4_O_3_ 409.1670, found 409.1669.

#### 4.1.3. 1-cyclopropyl-6-fluoro-7-(1-methyl-1H-imidazol-4-yl)-4-oxo-1,4-dihydroquinoline-3-carboxylic acid (**7**)

A mixture of 1 (150 mg, 0.567 mmol), methylimidazole (0.113 mL, 1.41 mmol) and 8.5 mL of anhydrous CH_3_CN was refluxed with drying tube. After 48 h, the resulting mixture was filtered and the solid was washed with warm acetonitrile (3 × 3 mL). After drying the product under vacuum, 154 mg of beige solid was obtained (yield: 83%; mp: 266 °C) IR (cm^−1^): 3174.5, 3117.00, 3042.88, 1726.88, 1618.17, 1544.72, 1488.95. NMR ^1^H (300 Hz, D_2_O) δ 8.69 (s, 1H), 8.45 (s, 1H), 8.09 (d, *J* = 9.0 Hz, 1H), 7.94 (s, 1H), 7.70 (s, 1H), 4.04 (s, 3H), 3.81–3.57 (m, 1H), and 1.44–1.01 (m, 4H). HRMS (ESI-TOF) *m*/*z*: (M + H)^+^ calculated for C_17_H_14_FN_3_O_3_ 328.1092, found 328.1099.

### 4.2. Antibiotic Activity (MIC and MBC)

All compounds were evaluated for their in-vitro MIC and MBC using standard techniques, following the protocol recommended by CLSI guidelines [33]. The synthesized compounds were dried before preparing stock solutions by keeping them in a vacuum in presence of P_2_O_5_ for at least 12 h. From the stock solutions, two-fold serial dilutions were prepared. Ciprofloxacin was used as a reference in order to compare MIC values. All experiments were repeated five times to ensure reproducibility. Activity values are provided as modal MICs and MBCs.

The strains used to perform the antibiotic activity assays are detailed in Table 6.

## 5. Conclusions

Currently, the development of antibiotic resistance is one of the biggest issues in the treatment of bacterial infections. Molecular topology, along with linear discriminant analysis, have proven to be useful tools in the design and search for new drugs. The present prediction model identified two quinolones with good antibacterial activity against *E. coli* and other bacteria. Furthermore, the knowledge and study of organic reactions has also resulted in the finding of a new type of zwitterionic quinolonate with antibacterial activity. These compounds can lead to a new line of antimicrobials, since their structures, and, therefore, their properties, are easily adjustable in the amine in position 4 of the pyridine ring.

## Figures and Tables

**Table 1:** Quinolone obtention from difluorinated quinolone **1**.

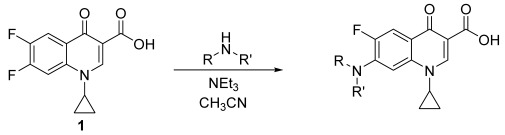							
Entry	Amine	eq.	Et_3_N (eq.)	Catalyst	Time	Quinolone	Yield
1	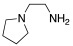	4	-	-	3 h	2	60%
2	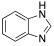	2	3	-	240 h	3	18%
3	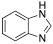	2	3	DMAP 20% molar	240 h	3	54%
4	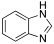	2	3	PBu_3_ 20% molar	240 h	3	17%

**Table 2:** Reaction of **1** with DMAP derivatives and 1-methylimidazole.

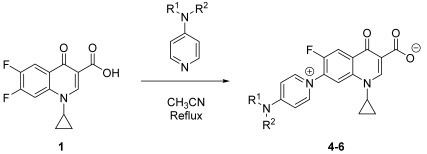					
Entry	Amine	eq.	Time	Product	Yield
1	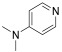	2.5	3 h	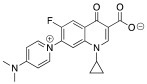 **4**	99%
2	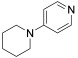	3.5	5 h	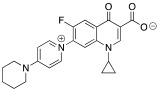 **5**	99%
3	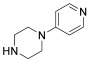	2.5	4 h	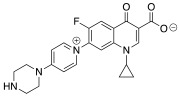 **6**	56%

**Table 3:** MIC and MBC values of the newly synthesized quinolones against *E. coli*.

Compound	MIC (mg/L)	MBC (mg/L)
**2**	1	>128
**3**	1	32
**Ciprofloxacin**	0.25	64

**Table 4:** Antimicrobial activity spectrum of compounds 2 and 3.

Microorganism	Compound 2		Compound 3	
	MIC (mg/L)	MBC (mg/L)	MIC (mg/L)	MBC (mg/L)
MRSA	4	32	0.5	32
*Staphylococcus aureus*	2	32	0.25	64
*Streptococcus agalactiae*	-	-	0.12	32
*Bacillus subtilis*	0.25	1	<0.03	0.06
*Enterococcus faecalis*	4	>128	64	>128
*Pseudomonas aeruginosa*	32	64	1	128
*Serratia marcescens*	-	-	32	128
*Candida albicans*	32	128	128	>128

**Table 5:** Antibacterial activity spectrum of the zwitterionic quinolonates and the quinolone **7** (mg/L).

Compound	*S. aureus*		*S. agalactiae*		*S. marcescens*		*E. coli*	
	MIC	MBC	MIC	MBC	MIC	MBC	MIC	MBC
**4**	32	>512	32	>512	128	>512	8	256
**5**	32	>512	32	512	512	>512	128	512
**6**	32	>512	>512	>512	>512	>512	512	>512
**7**	>512	>512	>512	>512	128	>512	64	>512

**Table 6:** Microorganisms used in the antimicrobial activity assays.

Microorganism	Strain
Methicillin-resistant *Staphylococcus aureus*	CECT 5190
*Staphylococcus aureus*	CECT 239
*Streptococcus agalactiae*	CECT 183
*Bacillus subtilis*	CECT 39
*Enterococcus faecalis*	CECT 481
*Escherichia coli*	CECT 4972
*Pseudomonas aeruginosa*	CECT 110
*Serratia marcescens*	CECT 846
*Candida albicans*	CECT 1394

## Data Availability

Data is contained within the article and supplementary material.

## Supplementary Materials

The following are available online at https://www.mdpi.com/article/10.3390/ph15070818/s1, Figure S1: Virtual combinatorial library, Table S1: theoretically active compounds vs. *E. coli*. Figure S2. ^1^H NMR (300 MHz, CDCl3) of **2**, Figure S3. HRMS (ESI-TOF) *m*/*z* of **2**, Figure S4. ^1^H NMR (300 MHz, DMSO-*d_6_*) of **3**, Figure S5. HRMS (ESI-TOF) *m*/*z* of **3**, Figure S6. ^1^H NMR (300 MHz, CD_3_OD) of **4**, Figure S7. HRMS (ESI-TOF) *m*/*z* of **4**, Figure S8. ^1^H NMR (300 MHz, CD_3_OD) of **5**, Figure S9. HRMS (ESI-TOF) *m*/*z* of **5**, Figure S10. ^1^H NMR (300 MHz, D_2_O) of **6**, Figure S11. HRMS (ESI-TOF) *m*/*z* of **6**, Figure S12. ^1^H NMR (300 MHz, D_2_O) of **7**, Figure S13. HRMS (ESI-TOF) *m*/*z* of **7**.
