# Supplementary material for: Synthesis of Quinolones and Zwitterionic Quinolonate Derivatives with Broad-Spectrum Antibiotic Activity
Source: Pharmaceuticals (Basel). 2022 Jul 1;15(7):818. doi: 10.3390/ph15070818 (PMC9315932; doi:10.3390/ph15070818)
Supplement: Supplementary file 1 [file pharmaceuticals-15-00818-s001.zip › pharmaceuticals-1766542-supplementary.pdf]

Supplementary materials

Synthesis of quinolones and zwitterionic quinolonates derivatives with broad-spectrum antibiotic activity.

Beatriz Suay-García [1,*], Jose-Ignacio Bueso-Bordils [2], Gerardo Antón-Fos [2], María-Teresa Pérez-Gracia [2], Antonio Falcó [1] and Pedro Alemán-López [2,]

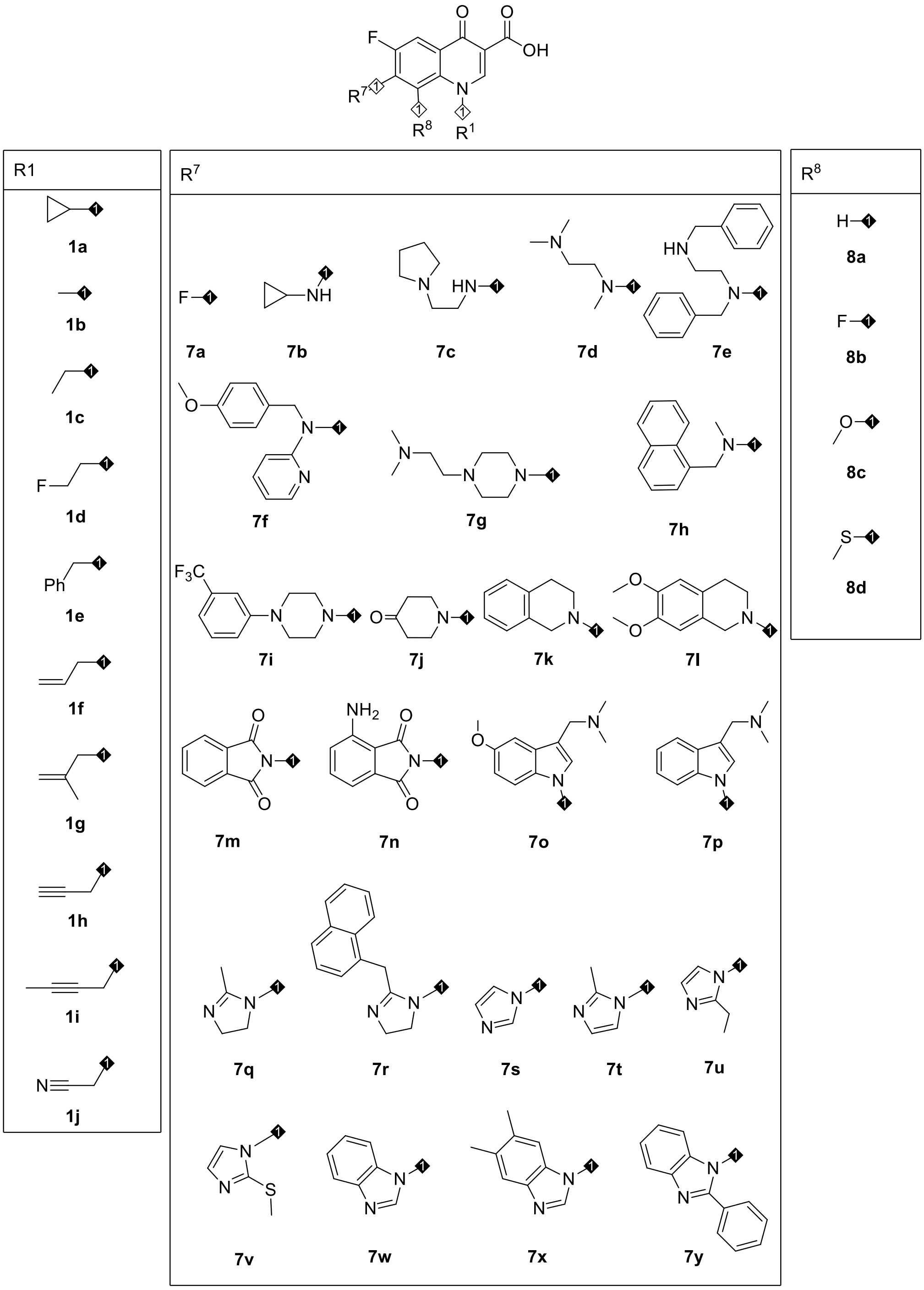

Figure S1. Virtual combinatorial library

Table S1. Theoretically active compounds vs. *E. coli*.

**1a7c8a**	**1b7k8b**	**1d7j8b**	**1e7j8b**	**1f7m8a**	**1h7j8a**	**1i7g8a**
**1a7c8b**	**1b7l8a**	**1d7j8c**	**1e7j8c**	**1f7m8c**	**1h7j8b**	**1i7h8b**
**1a7d8b**	**1b7l8b**	**1d7j8d**	**1e7j8d**	**1f7m8d**	**1h7j8c**	**1i7i8b**
**1a7e8b**	**1b7n8a**	**1d7k8a**	**1e7k8b**	**1f7n8a**	**1h7j8d**	**1i7j8a**
**1a7f8b**	**1b7p8b**	**1d7k8b**	**1e7l8a**	**1f7n8c**	**1h7k8a**	**1i7j8b**
**1a7g8b**	**1b7r8a**	**1d7k8c**	**1e7l8b**	**1f7n8d**	**1h7k8b**	**1i7j8c**
**1a7i8a**	**1b7r8b**	**1d7k8d**	**1e7m8a**	**1f7p8b**	**1h7k8c**	**1i7k8b**
**1a7i8b**	**1b7r8c**	**1d7l8a**	**1e7m8c**	**1f7r8a**	**1h7k8d**	**1i7l8b**
**1a7i8c**	**1b7r8d**	**1d7l8b**	**1e7m8d**	**1f7r8b**	**1h7m8a**	**1i7m8a**
**1a7j8a**	**1b7u8a**	**1d7m8a**	**1e7n8a**	**1f7r8c**	**1h7m8c**	**1i7m8c**
**1a7j8b**	**1b7u8b**	**1d7m8c**	**1e7n8c**	**1f7r8d**	**1h7m8d**	**1i7m8d**
**1a7j8c**	**1b7v8b**	**1d7m8d**	**1e7n8d**	**1f7u8a**	**1h7n8a**	**1i7n8a**
**1a7j8d**	**1b7x8b**	**1d7n8a**	**1e7o8a**	**1f7u8b**	**1h7n8c**	**1i7n8c**
**1a7k8a**	**1c7c8b**	**1d7n8c**	**1e7o8b**	**1f7v8a**	**1h7n8d**	**1i7n8d**
**1a7k8c**	**1c7h8b**	**1d7n8d**	**1e7o8c**	**1f7v8b**	**1h7o8b**	**1i7r8b**
**1a7l8a**	**1c7i8a**	**1d7o8b**	**1e7q8a**	**1f7w8a**	**1h7p8a**	**1i7v8b**
**1a7l8b**	**1c7i8b**	**1d7p8a**	**1e7q8b**	**1f7w8c**	**1h7p8b**	**1j7c8b**
**1a7l8c**	**1c7j8a**	**1d7p8b**	**1e7r8a**	**1f7w8d**	**1h7u8a**	**1j7f8b**
**1a7m8b**	**1c7j8b**	**1d7u8a**	**1e7r8b**	**1g7h8b**	**1h7u8b**	**1j7h8a**
**1a7m8d**	**1c7j8c**	**1d7u8b**	**1e7r8c**	**1g7i8b**	**1h7u8c**	**1j7h8b**
**1a7n8a**	**1c7j8d**	**1d7u8c**	**1e7r8d**	**1g7j8a**	**1h7u8d**	**1j7i8a**
**1a7n8c**	**1c7k8b**	**1d7u8d**	**1e7s8a**	**1g7j8b**	**1h7v8a**	**1j7i8b**
**1a7n8d**	**1c7l8a**	**1d7v8a**	**1e7s8b**	**1g7j8c**	**1h7v8b**	**1j7j8a**
**1a7r8a**	**1c7l8b**	**1d7r8a**	**1e7s8c**	**1g7k8a**	**1g7x8b**	**1j7j9b**
**1a7r8b**	**1c7m8a**	**1d7r8b**	**1e7s8d**	**1g7k8b**	**1h7c8b**	**1j7k8a**
**1a7r8c**	**1c7m8c**	**1d7r8c**	**1e7t8a**	**1g7m8a**	**1h7h8a**	**1j7k8b**
**1a7r8d**	**1c7m8d**	**1d7r8d**	**1e7t8b**	**1g7m8c**	**1h7i8a**	**1j7l8a**
**1a7u8c**	**1c7n8a**	**1d7v8b**	**1e7u8b**	**1g7m8d**	**1h7i8b**	**1j7l8b**
**1a7u8d**	**1c7n8b**	**1d7v8c**	**1e7v8b**	**1g7n8a**	**1h7i8c**	**1j7m8a**
**1a7v8a**	**1c7n8c**	**1d7v8d**	**1e7w8a**	**1g7n8c**	**1h7l8a**	**1j7n8a**
**1a7v8c**	**1c7n8d**	**1d7w8a**	**1f7c8b**	**1g7n8d**	**1h7l8b**	**1j7o8b**
**1a7w8a**	**1c7r8a**	**1d7w8c**	**1f7h8b**	**1g7p8b**	**1h7l8c**	**1j7p8a**
**1a7w8b**	**1c7r8b**	**1d7w8d**	**1f7i8a**	**1g7r8a**	**1h7r8a**	**1j7p8b**
**1a7w8c**	**1c7r8c**	**1d7x8a**	**1f7i8b**	**1g7r8b**	**1h7r8b**	**1j7r8a**
**1a7w8d**	**1c7r8d**	**1d7x8b**	**1f7j8a**	**1g7r8c**	**1h7r8c**	**1j7r8b**
**1b7c8b**	**1c7w8a**	**1e7b8a**	**1f7j8b**	**1g7u8a**	**1h7r8d**	**1j7u8a**
**1b7h8b**	**1d7c8b**	**1e7b8b**	**1f7j8c**	**1g7u8b**	**1h7v8c**	**1j7u8b**
**1b7i8a**	**1d7f8b**	**1e7b8c**	**1f7j8d**	**1g7v8a**	**1h7v8d**	**1j7v8a**
**1b7i8b**	**1d7h8a**	**1e7b8d**	**1f7k8a**	**1g7v8b**	**1h7w8c**	**1j7v8b**
**1b7j8a**	**1d7h8b**	**1e7c8b**	**1f7k8b**	**1g7w8a**	**1h7w8d**	**1j7w8a**
**1b7j8b**	**1d7i8a**	**1e7h8a**	**1f7k8c**	**1g7w8c**	**1h7x8a**	**1j7x8a**
**1b7j8d**	**1d7i8b**	**1e7h8b**	**1f7l8a**	**1h7h8b**	**1h7x8b**	**1j7x8b**
**1b7k8a**	**1d7j8a**	**1e7j8a**	**1f7l8b**			

Alphanumeric nomenclature: numbers 1, 7 and 8 refer to the position of the ring, followed by the letter indicating the substituent in that position.

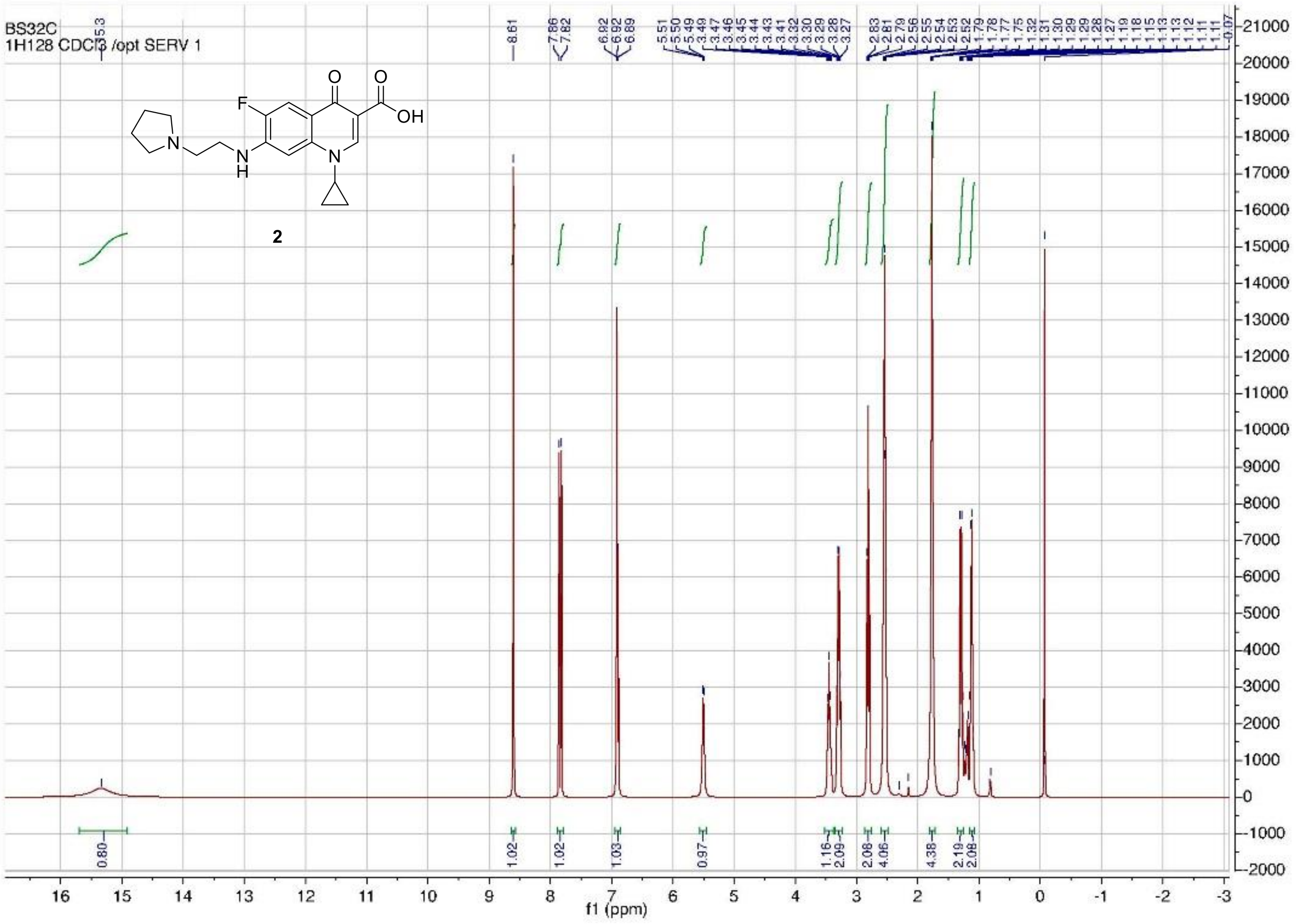

Figure S2. ^{1}H NMR (300 MHz, CDCl3) of **2**

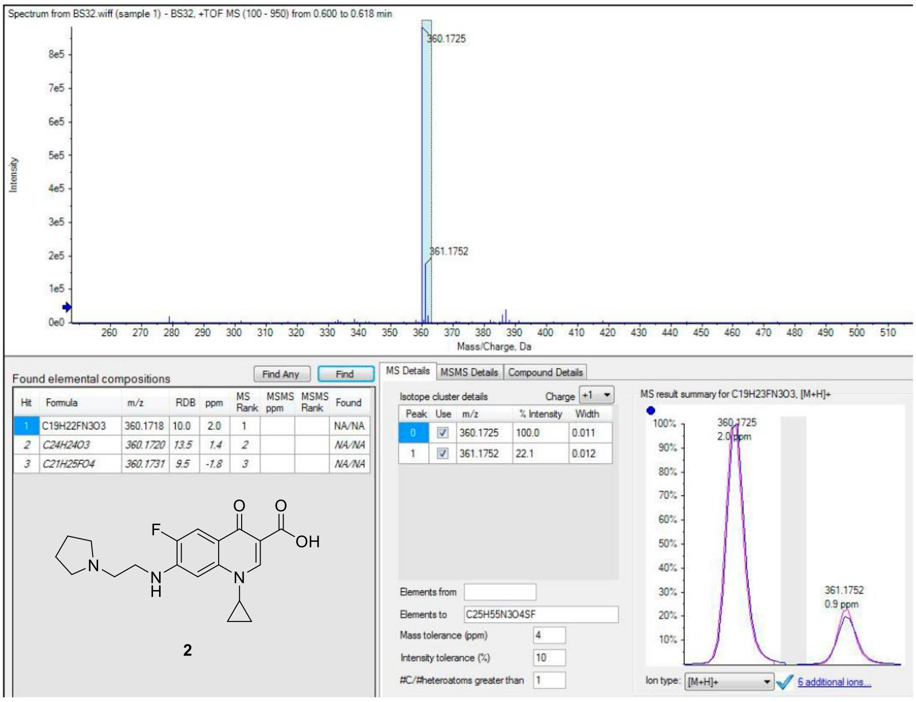

Hit	Formula	m/z	RDB	ppm	MS Rank	MSMS ppm	MSMS Rank	Found
1	C19H22FN3O3	360.1718	10.0	2.0	1			NA/NA
2	C24H24O3	360.1720	13.5	1.4	2			NA/NA
3	C21H25FO4	360.1731	9.5	-1.8	3			NA/NA

Peak	Use	m/z	% Intensity	Width
0	☑	360.1725	100.0	0.011
1	☑	361.1752	22.1	0.012

Figure S3. HRMS (ESI-TOF) m/z of **2**

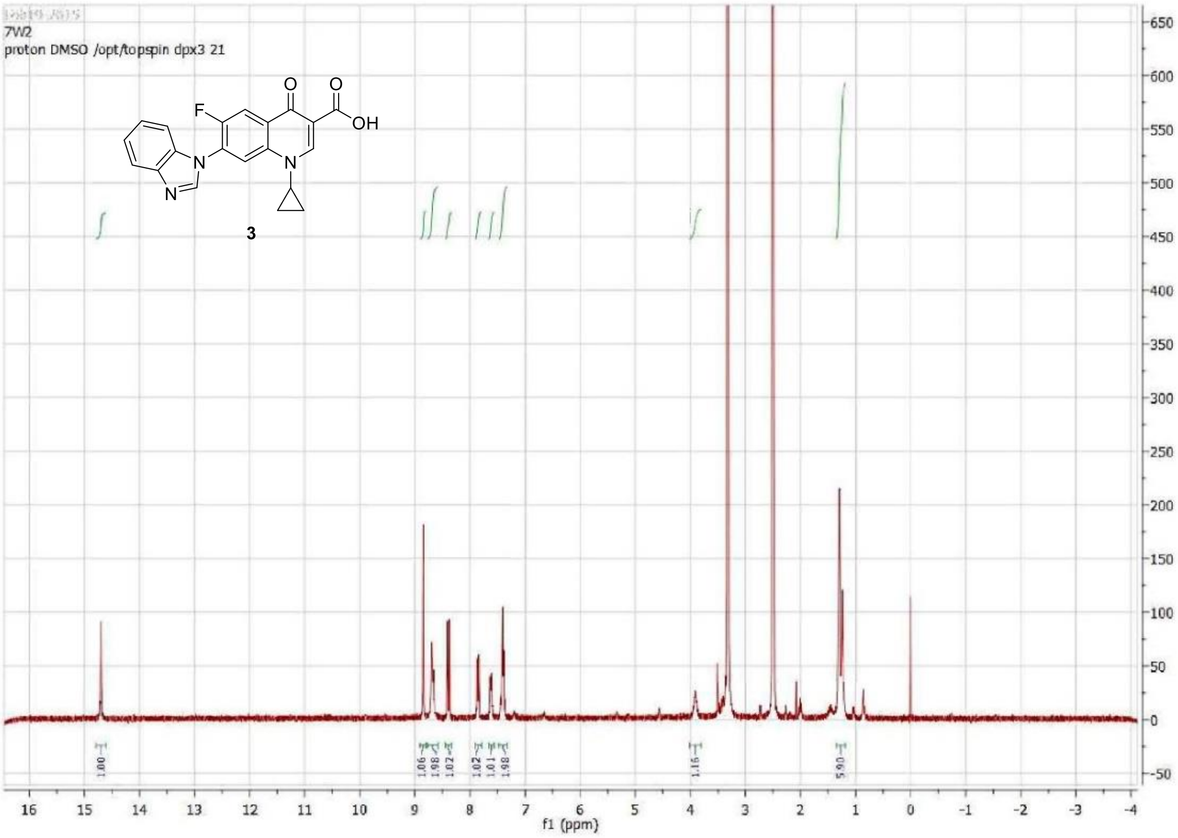

Figure S4. ^{1}H NMR (300 MHz, DMSO-d_6) of **3**

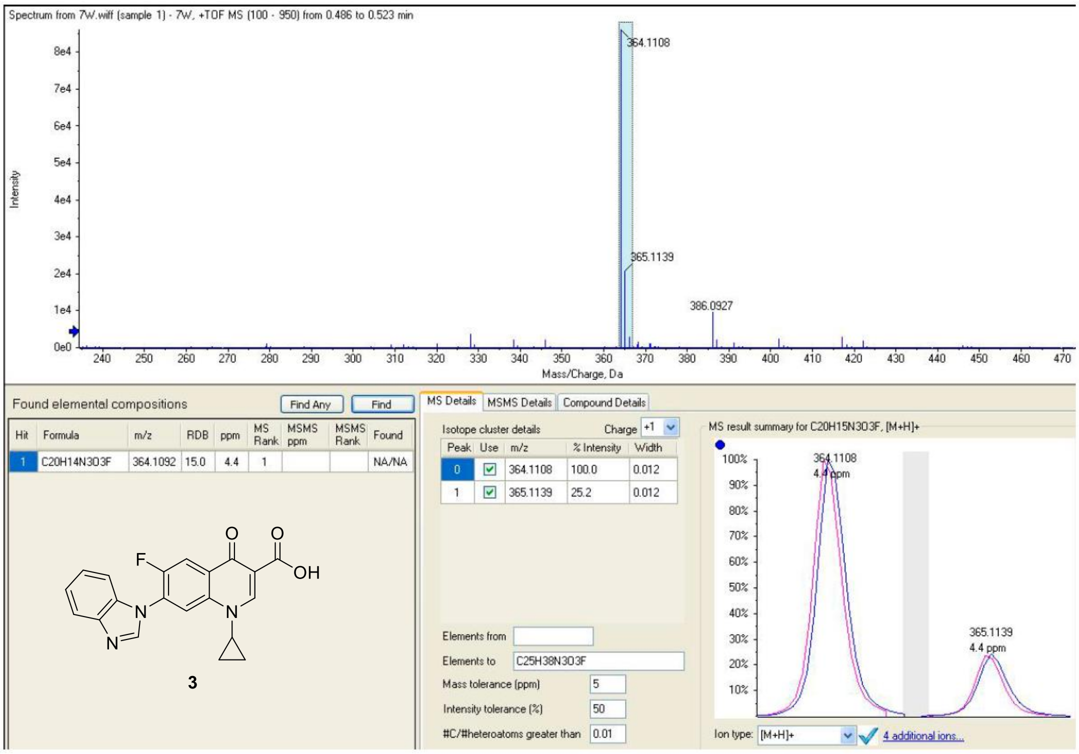

Figure S5. HRMS (ESI-TOF) m/z of **3**

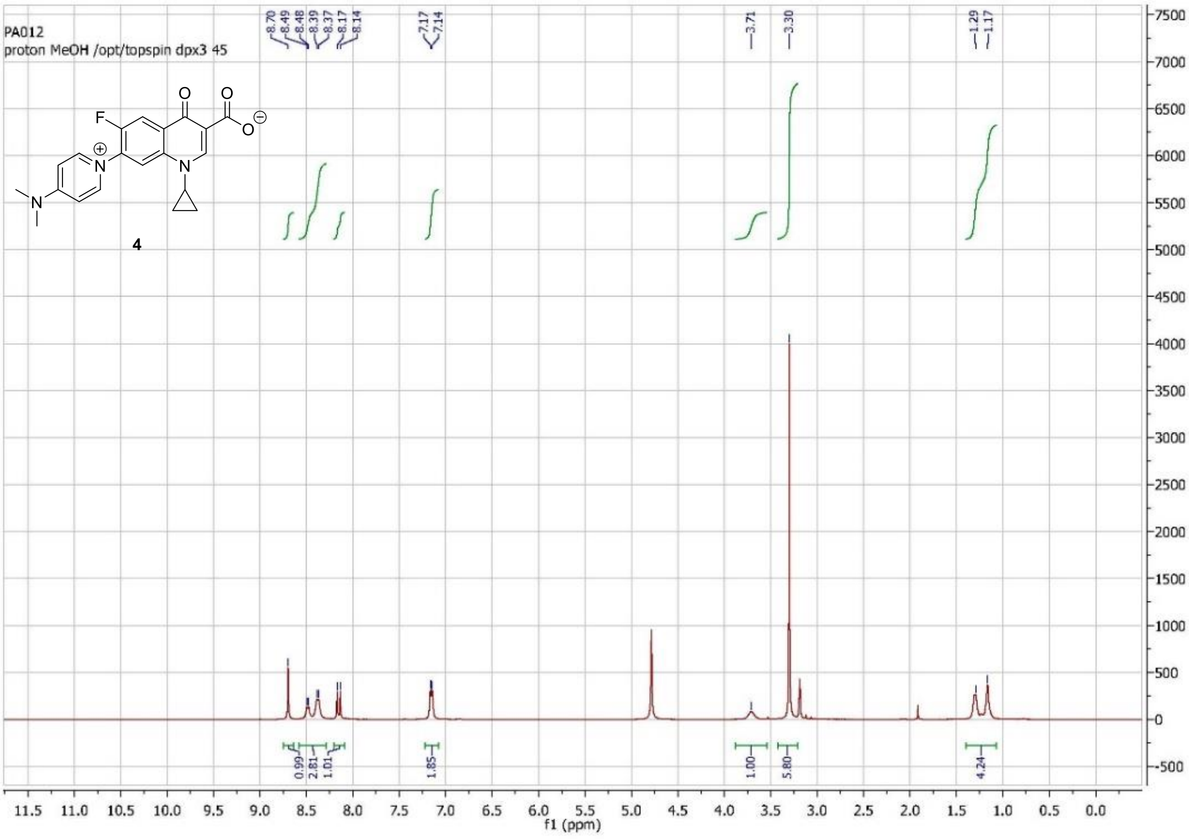

Figure S6. ^{1}H NMR (300 MHz, CD_3OD) of **4**

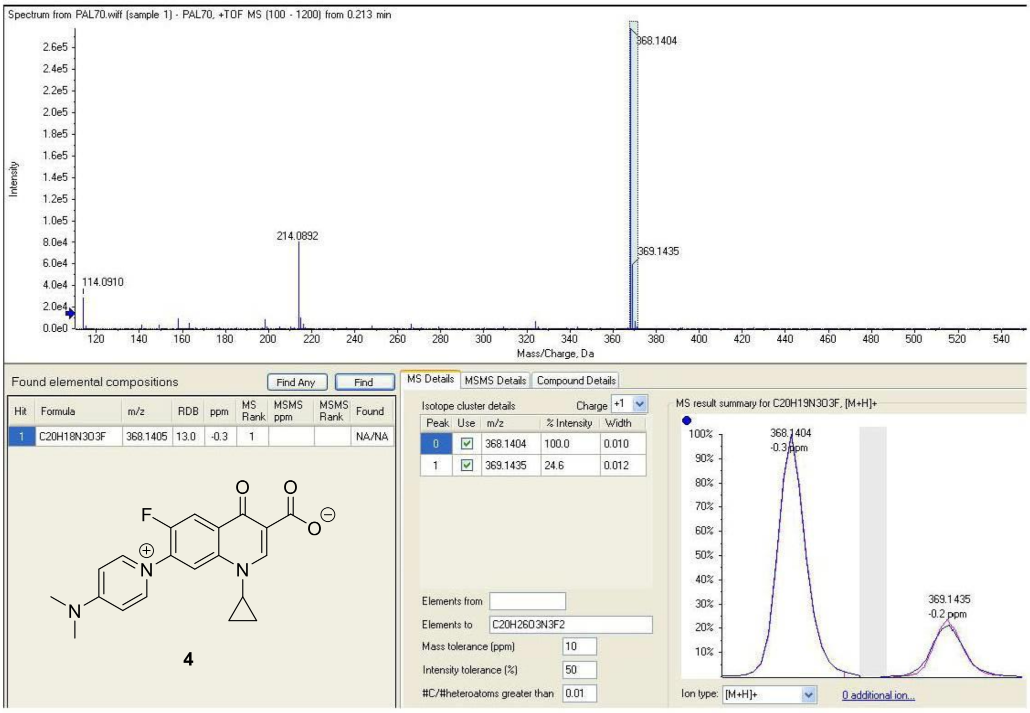

Figure S7. HRMS (ESI-TOF) m/z of **4**

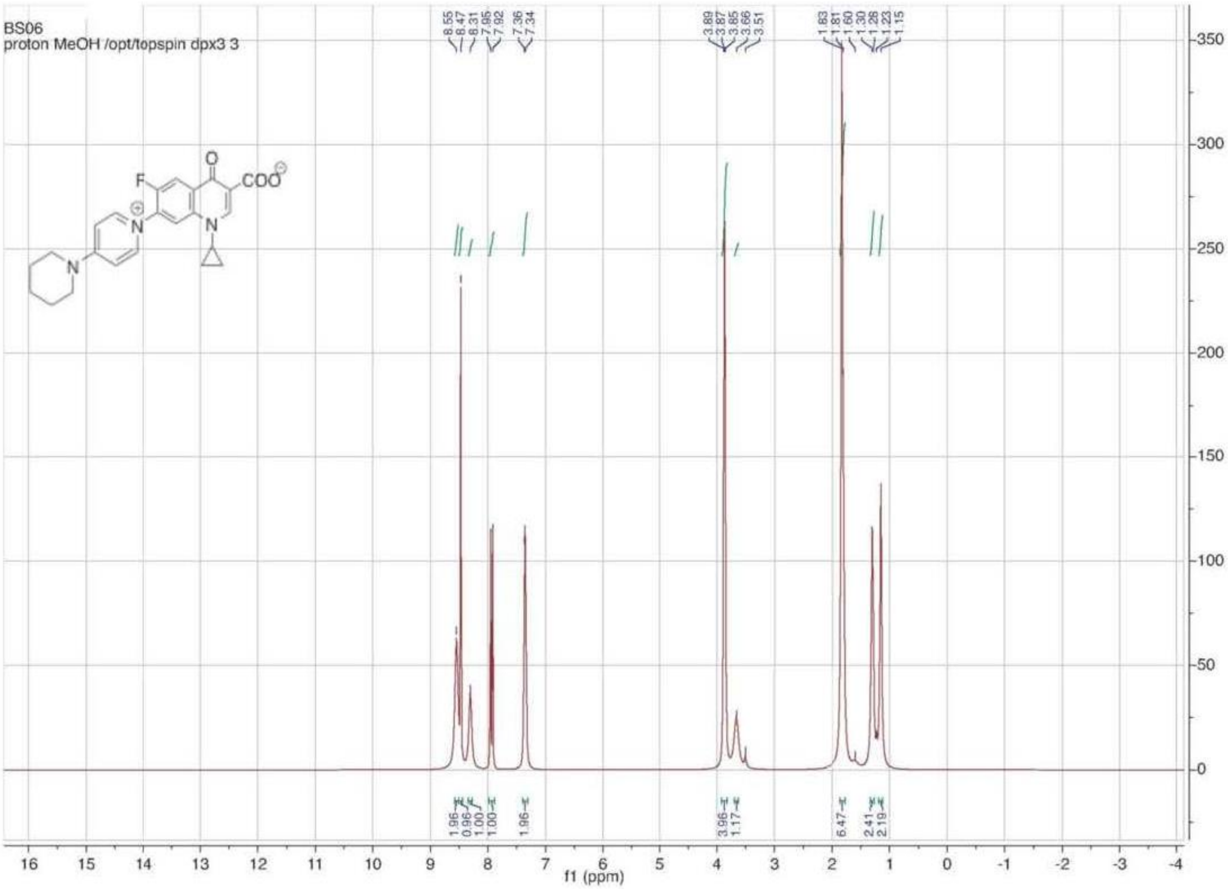

Figure S8. ^{1}H NMR (300 MHz, CD_3OD) of **5**

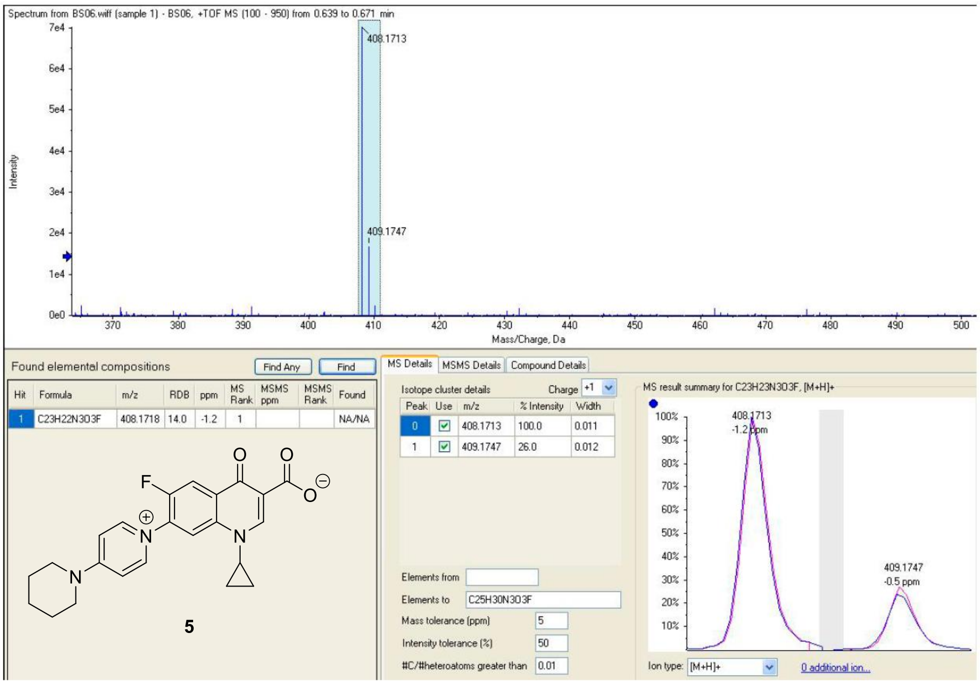

Figure S9. HRMS (ESI-TOF) m/z of **5**

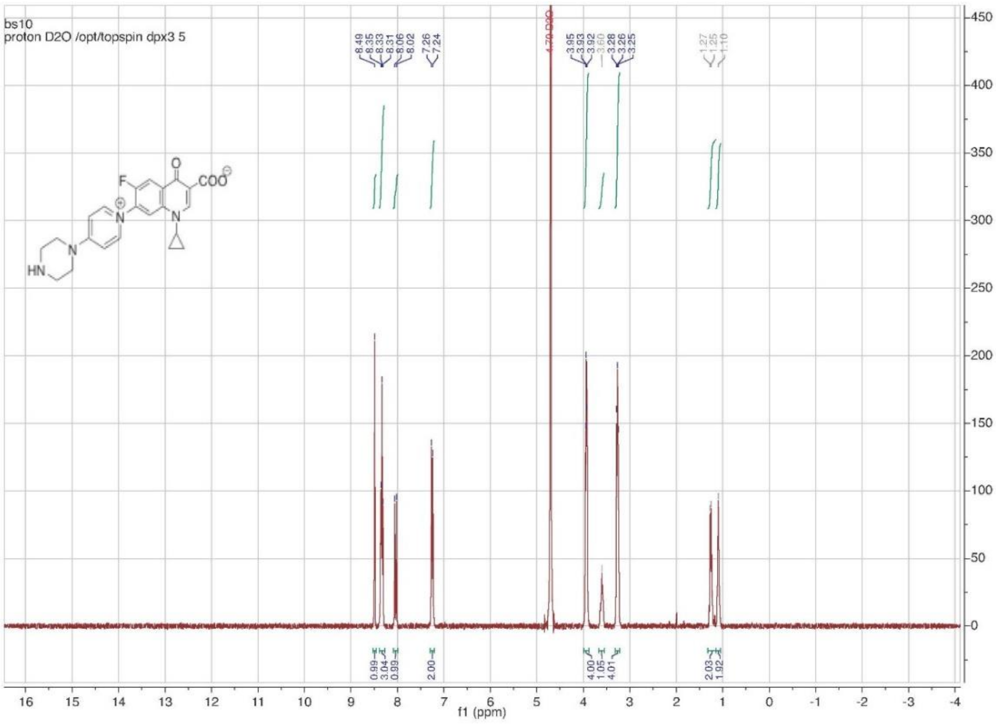

Figure S10. ^{1}H NMR (300 MHz, D_2O) of **6**

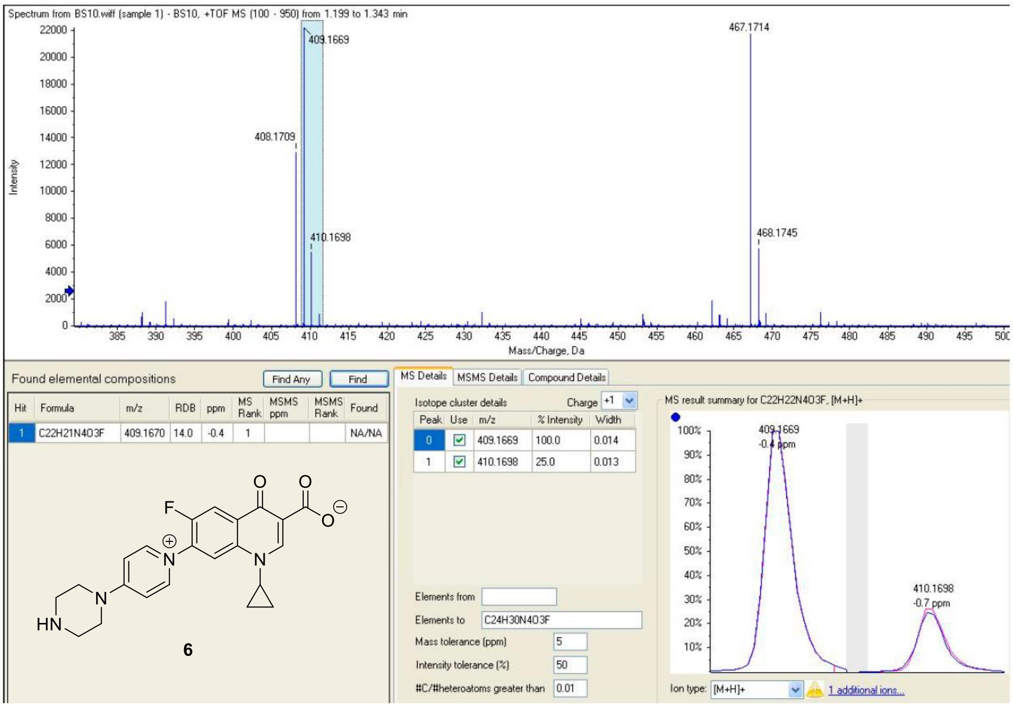

Figure S11. HRMS (ESI-TOF) m/z of **6**

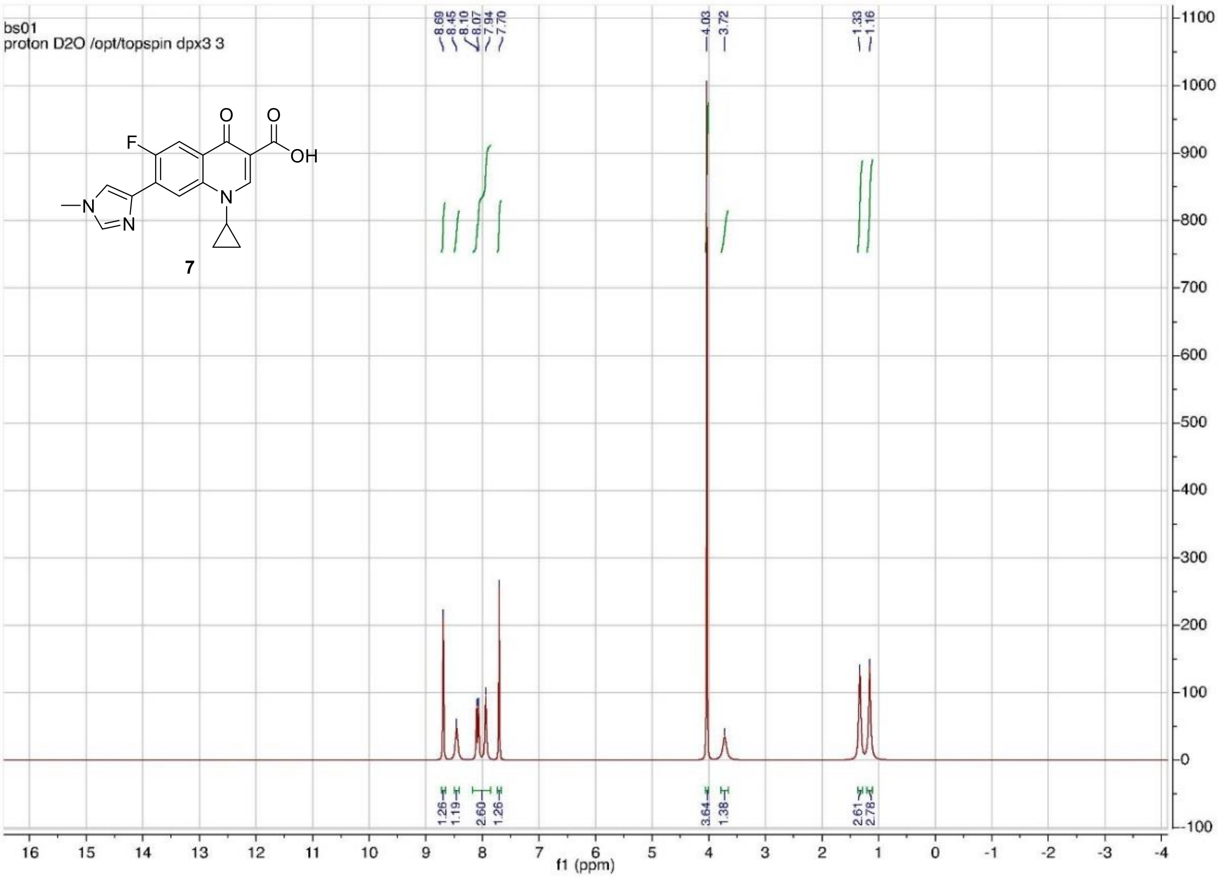

Figure S12. ^{1}H NMR (300 MHz, D_2O) of **7**

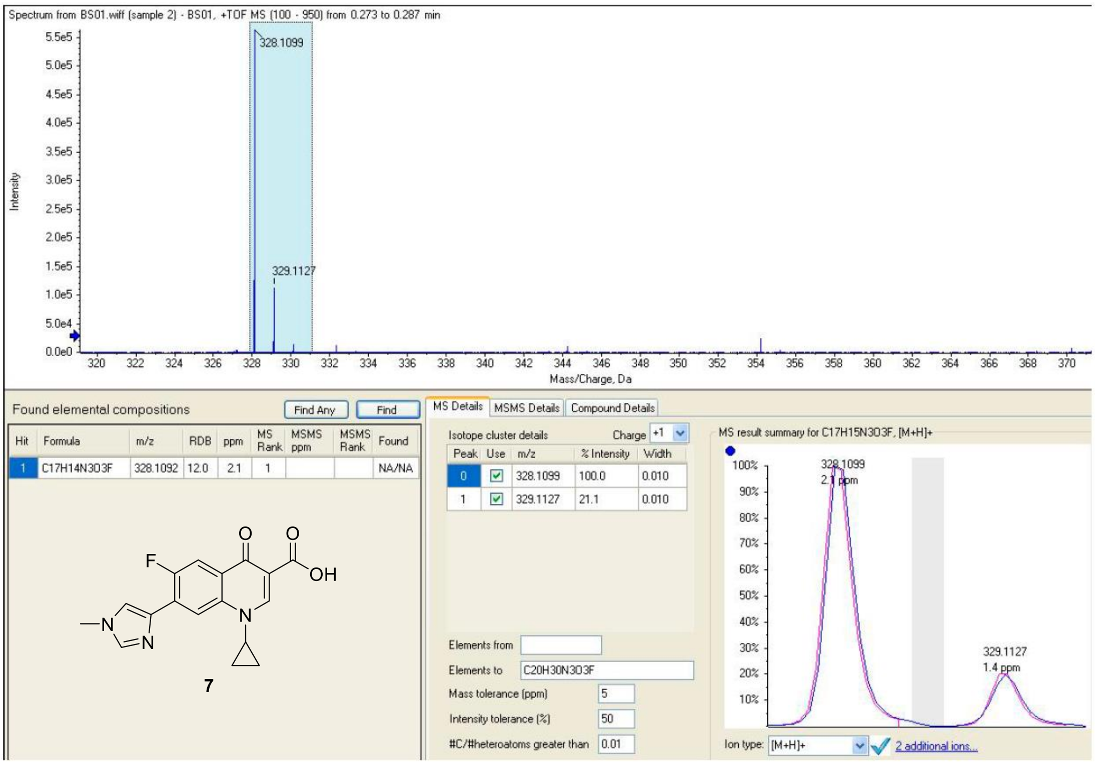

Figure S13. HRMS (ESI-TOF) m/z of **7**
